# Pyrene conjugation and spectroscopic analysis of hydroxypropyl methylcellulose compounds successfully demonstrated a local dielectric difference associated with *in vivo* anti-prion activity

**DOI:** 10.1371/journal.pone.0185357

**Published:** 2017-09-21

**Authors:** Kenta Teruya, Ayumi Oguma, Keiko Nishizawa, Hiroshi Kamitakahara, Katsumi Doh-ura

**Affiliations:** 1 Department of Neurochemistry, Tohoku University Graduate School of Medicine, Sendai, Miyagi, Japan; 2 Division of Forest and Biomaterials Science, Kyoto University Graduate School of Agriculture, Kyoto, Kyoto, Japan; National Institute of Allergy and Infectious Diseases, UNITED STATES

## Abstract

Our previous study on prion-infected rodents revealed that hydroxypropyl methylcellulose compounds (HPMCs) with different molecular weights but similar composition and degree of substitution have different levels of long-lasting anti-prion activity. In this study, we searched these HPMCs for a parameter specifically associated with *in vivo* anti-prion activity by analyzing *in vitro* chemical properties and *in vivo* tissue distributions. Infrared spectroscopic and thermal analyses revealed no differences among HPMCs, whereas pyrene conjugation and spectroscopic analysis revealed that the fluorescence intensity ratio of peak III/peak I correlated with anti-prion activity. This correlation was more clearly demonstrated in the anti-prion activity of the 1-year pre-infection treatment than that of the immediate post-infection treatment. In addition, the intensity ratio of peak III/peak I negatively correlated with the macrophage uptake level of HPMCs in our previous study. However, the *in vivo* distribution pattern was apparently not associated with anti-prion activity and was different in the representative tissues. These findings suggest that pyrene conjugation and spectroscopic analysis are powerful methods to successfully demonstrate local dielectric differences in HPMCs and provide a feasible parameter denoting the long-lasting anti-prion activity of HPMCs *in vivo*.

## Introduction

Prion diseases, including Creutzfeldt–Jakob disease in humans, are progressive fatal neurodegenerative illnesses [1] characterized by the pathogenic accumulation of abnormal prion proteins, which are the primary components of the infectious agent called a prion. Prions conformationally transform normal proteins into abnormal proteins in the central nervous and lymphoreticular systems [2]. Several compounds have been reported to prolong incubation periods in prion-infected animals [3–5]; however, sufficiently beneficial effects in patients have never been reported [6, 7]. This is partly because human prion strains may react differently to the compounds [8] and partly because most prion diseases in humans occur in a subacute progressive fashion, making it difficult to intervene in a timely manner [7]. Therefore, preventive interventions and preclinical or early diagnosis, as well as elucidation of efficacy against human prion strains, are now considered necessary for significantly beneficial outcomes in humans.

Our previous study demonstrated that a single administration of hydroxypropyl methylcellulose compounds (HPMCs) can dose-dependently prolong the survival of prion disease model rodents [9]. The most remarkable effectiveness was observed when HPMCs were administered immediately after prion infection; a similar level of effectiveness was also observed when administered 1 year prior to infection. Interestingly, the prolonged survival of prion-infected rodents was related to the viscosity (molecular size) of HPMCs with a similar composition and degree of substitution. The relationship between the efficacy and viscosity of these HPMCs exhibited a bell-type profile of the logarithm of viscosity. HPMCs inhibited prion accumulation in a similar viscosity-related manner in persistently prion-infected cells and also inhibited *in vitro* prion amplification reactions [9]. However, it is still unclear what kinds of chemical properties of HPMCs are specifically associated with anti-prion activity.

In this study, we searched for a parameter associated with the long-lasting *in vivo* anti-prion activity of HPMCs by analyzing both *in vitro* chemical properties and *in vivo* tissue distributions of the same HPMCs that were used in our previous *in vivo* study [9]. These HPMCs have different number-averaged molecular weights (*M*_n_) but a similar composition and degree of substitution. The *in vitro* chemical properties were evaluated using infrared spectroscopic, thermal, and pyrene conjugation spectroscopic analyses, while the *in vivo* tissue distributions were evaluated in the representative tissues using fluorescein-labeled HPMCs.

## Materials and methods

### Ethics statement

All animal experiments were performed in accordance with protocols reviewed and approved by the Institutional Animal Care and Use Committee of Tohoku University (approval number 21MdA192). The animal care and use protocols adhered to the Fundamental Guidelines for Proper Conduct of Animal Experiment and Related Activities in Academic Research Institutions by the Ministry of Education, Culture, Sports, Science and Technology (Notice No. 71, issued June 1, 2006), the Standards Relating to the Care and Management of Laboratory Animals and Relief of Pain by the Ministry of the Environment (Notice No. 84, issued August 30, 2013), and the Act on Welfare and Management of Animals (revised September 5, 2012) in Japan.

### HPMCs

All HPMCs used in the study are type E (hypromellose 2910) classified by the United States Pharmacopeia [10] and were kindly provided by Shin-Etsu Chemical Co., Ltd. (Tokyo, Japan). The data of composition and degree of substitution and viscosity, as well as the product name were provided by Shin-Etsu Chemical. All other data in Table 1 were obtained from our previous report [9], including the calculated *M*_n_, the macrophage uptake level, and the median survival time in prion-infected Tg7 mice (% of vehicle control) immediately post-infection or 1-year pre-infection treatments.

**Table 1:** Chemical and biological properties of HPMCs.

	Degree of substitution		Viscosity (2%, 20°C, mPas)	Molecular weight (*M*_n_) [9]	Macrophage uptake level (‰) [9]	Median survival time in prion infection (% of vehicle) [9] *^1^	
	O-CH_3_	O-CH_2_CH(OH)CH_3_				Immediate post-infection treatment	1-year pre-infection treatment
HPMC602	1.90	0.24	1.7	7,215	0.158	146 [146–146]	ND *^2^
TC-5RW	1.91	0.24	5.8	10,695	0.171	268 [247–291]	156 [153–180]
60SH-50	1.88	0.24	50.4	21,257	0.128	324 [265–417]	222 [191–357]
60SH-400	1.92	0.24	390	40,720	0.083	310 [287–323]	316 [227–352]
60SH-4K	1.92	0.24	3860	84,351	0.149	236 [222–246]	179 [178–183]
60SH-10K	1.92	0.24	9730	113,150	0.184	196 [188–211]	168 [160–190]

*^1^ Data of 1st and 3rd quartiles in parentheses

*^2^ No data

### Fourier-transform infrared spectroscopy (FTIR) measurement

HPMC films for FTIR measurement were prepared by casting 500 μL of each HPMC solution (10 mg/mL in water) on a 2 × 2 × 1 cm polystyrene balance dish and subsequently air drying at room temperature. FTIR-attenuated total reflectance (ATR) spectra were measured on a Perkin Elmer 100 Fourier spectrometer (PerkinElmer, Inc., USA) equipped with a universal ATR sampling accessory. Measurement was performed at a 4 cm^−1^ resolution in 380–4000 cm^−1^ at room temperature, and data from over 16 scans were averaged.

### Differential scanning calorimetry (DSC) measurement

DSC thermograms were recorded on a DSC823e (Mettler Toledo, Switzerland) with a MultiSTAR HSS7 DSC sensor under a nitrogen (N_2_) atmosphere during a heating/cooling cycle (0°C→90°C→0°C) with a heating and cooling rate of 3.5°C/min. Each temperature cycle was sequentially repeated three times to ensure and check for reproducible responses of the instrument.

### Fluorescein labeling of HPMCs

HPMCs were dissolved in *N*,*N*-dimethylformamide (DMF) (0.3 g/30 mL) and mixed with 1.5 mg of fluorescein-5-carbonyl azide diacetate. The mixtures were purged with N_2_ gas and agitated at 90°C for 3 h. Excess fluorescein reagent was removed by repeated precipitation and solubilization steps; briefly, labeled HPMCs were recovered by the addition of hexane to the reaction mixture, and residual solids were redissolved in DMF. The resultant labeled HPMCs were dissolved in aqueous NaHCO_3_ solution (100 mM, pH 8.0) at room temperature for 8 h for the deprotection of acetyl groups according to the manufacturer’s instructions. The resulting solution was dialyzed against deionized water on an acetyl cellulose membrane (MW cut-off: 3,500 Da) for 2 days. Fluorescein-labeled HPMC solutions were filtered through a 0.45-μm-pore filter and lyophilized. No fluorescent signal was detected in low molecular weight fractions by gel permeation chromatography (GPC) analysis using a fluorescence detector (column, OHPak SB-804 HQ [Showa Denko, Tokyo, Japan]; detection, excitation at 494 nm and recording at 520 nm).

### Pyrene conjugation and spectroscopic analysis

Pyrene conjugation to HPMCs was performed as follows: 1-pyrenebutyryl chloride in dichloromethane (10 mg/1.0 mL) was added to HPMCs in DMF (0.20 g/20 mL). The mixtures were purged with N_2_ gas and agitated at 60°C overnight. Pyrene-bound HPMCs were precipitated by adding 30 mL hexane and 10 mL chloroform to the reaction mixture. The resulting solids were recovered by centrifugation and redissolved in 20 mL DMF. This precipitation process was repeated twice. The resulting precipitant was washed with 5 mL hexane and dried. The white precipitant was then dissolved in 160 mL deionized water. The solution was dialyzed against deionized water using an acetyl cellulose membrane (MW cut-off: 3,500 Da) for 2 days. The pyrene-bound HPMC solution was filtered through a 0.45-μm-pore filter and lyophilized.

To measure the fine structure of pyrene fluorescence in pyrene-bound HPMC solutions (1.0 mg/mL in water), fluorescence spectra excited at 310 nm were recorded using a JASCO FP-6500 (Tokyo, Japan) with a scanning range of 350–500 nm and a data point interval of 0.2 nm.

### Tissue distribution analysis of HPMCs *in vivo*

Both ddY and ICR mice were purchased from Japan SLC, Inc. (Hamamatsu, Japan). Tg7 mice [11] were kindly provided by Dr. Bruce Chesebro of the Laboratory of Persistent Viral Diseases of NIAID’s Rocky Mountain Laboratories (Hamilton, MT, USA). Mice were maintained under specific pathogen-free conditions at about 24°C on a 12-h light cycle, with free access to water and standard diet throughout the experiments. Five-week old male mice were used for the experiments. For measuring fluorescein-labeled TC-5RW in the blood or plasma, three mice were intraperitoneally injected with 1 mL of fluorescein-labeled TC-5RW solution (50 mg/mL) or saline, and blood samples were collected from the mice under deep pentobarbital anesthesia 1 week after administration. The samples were mixed with heparin, and aliquots were centrifuged to obtain plasma components.

For measuring fluorescein-labeled HPMCs in brain choroid plexus and spleen, 1 mL of each fluorescein-labeled HPMC solution (50 mg/mL) or saline was intraperitoneally injected into three mice from each group. The animals were euthanized under deep pentobarbital anesthesia 1 week or 8 weeks after administration by perfusion using lactated Ringer’s solution containing 10% Coomassie Brilliant Blue. Subsequently, brain choroid plexus and spleen were carefully collected. Each collected tissue sample was put into a 2-mL tube containing ceramic beads and stored at −80°C until use. The tissue samples were homogenized with lysis buffer (phosphate buffer saline [PBS] containing 0.5% deoxycholate, 0.5% NP-40, and a protease inhibitor cocktail [Roche Diagnostic, Indianapolis, USA]) using a Precellys 24 homogenizer (Bertin Instruments, Paris, France); the homogenates were briefly centrifuged to remove tissue debris and the ceramic beads.

For each blood sample, 20 μL of whole blood or plasma was diluted by adding 180 μL of lysis buffer. After filtration through a 0.45-μm-pore filter, 50 μL of a 200-μL diluted sample was subjected to GPC analysis (column, OHPak SB-804 HQ [8.0 mm × 300 mm, Shodex]; eluent, PBS, 1.0 mL/min; detection, excitation at 494 nm and recording at 520 nm). For each brain choroid plexus sample, 30 μL of a 250-μL homogenate was subjected to GPC analysis. For each spleen sample, 40 μL of a 1000-μL homogenate was diluted by adding 160 μL of lysis buffer. After filtration through a 0.45-μm-pore filter, a 30-μL aliquot of the 200-μL diluted sample was subjected to GPC analysis. Standard samples of each fluorescein-labeled HPMC were prepared by serial dilution with lysis buffer. The amount of labeled HPMCs contained in each tissue sample was calculated by measuring the area of the peak corresponding to the compound in the GPC chart and standardizing with standard samples.

### Statistical analysis

Statistical linear correlation was evaluated using Pearson’s correlation coefficient. All analyses were performed using statistical analysis software from Social Survey Research Information Co. Ltd. (Tokyo, Japan).

## Results and discussion

Various types of HPMCs are manufactured from cellulose and are widely used as multiple purpose ingredients in foods, cosmetics, and pharmaceuticals. Individual consumption of HPMCs from foods is estimated in humans to be about 3 mg/day which has a theoretical safety factor of >100,000, but that of pharmaceuticals has never been evaluated [12]. Orally taken HPMCs are exclusively excreted in feces at 97% in humans after 3 repeated doses of 8.9 g [13] and 97–102% in rats after 5 repeated doses of 500 mg/kg body weight [14], suggesting almost no tendency of accumulation in tissues [12–14]. However, it is unclear how much amount of HPMCs accumulates in tissues of humans after life-long daily exposures to them.

**Fig 1:**
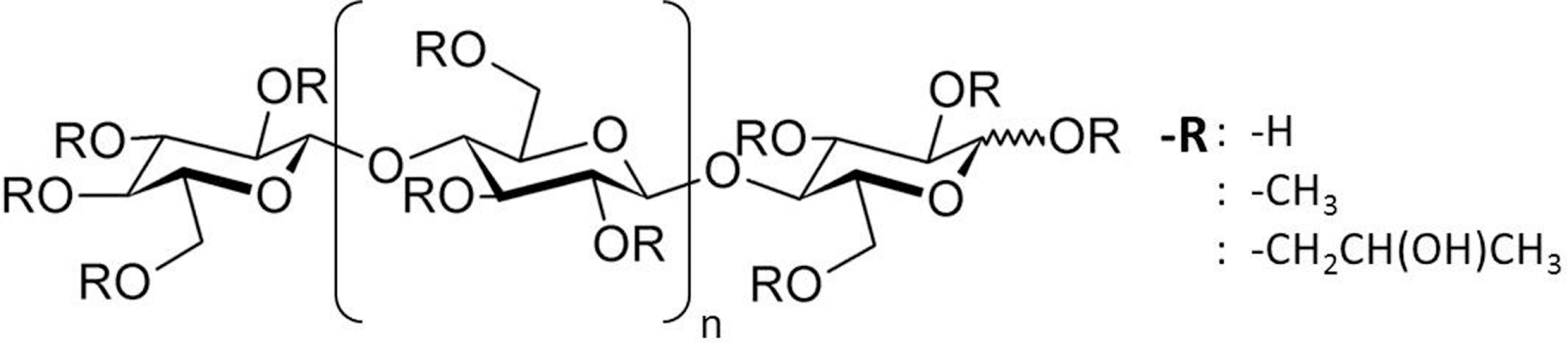
Chemical structure of HPMCs.

Cellulose is a natural polysaccharide that is insoluble in water because of the existence of strong intra- and inter-molecular interactions via hydrogen bonds [15]. In HPMCs (Fig 1), the replacement of hydroxyl groups by hydroxypropyl and methyl groups reduces hydrogen bonding and confers increased solubility in water [16]. Thus, the properties of HPMCs are characterized by their molar degree of substitution on the anhydroglucose unit and the degree of polymerization corresponding to *M*_n_.

In this study, we used two different strategies to investigate those HPMCs (hypromellose 2910) that are popularly used in the pharmaceutical industry and that are similar in composition and degree of substitution but are different in *M*_n_, macrophage uptake level, and anti-prion activity in prion-infected rodents as reported in our previous study [9] (Table 1). One strategy was an *in vitro* approach using non-labeled or fluorescent agent-labeled HPMCs, while the other followed an *in vivo* approach using fluorescent agent-labeled HPMCs.

### FTIR analysis

**Fig 2:**
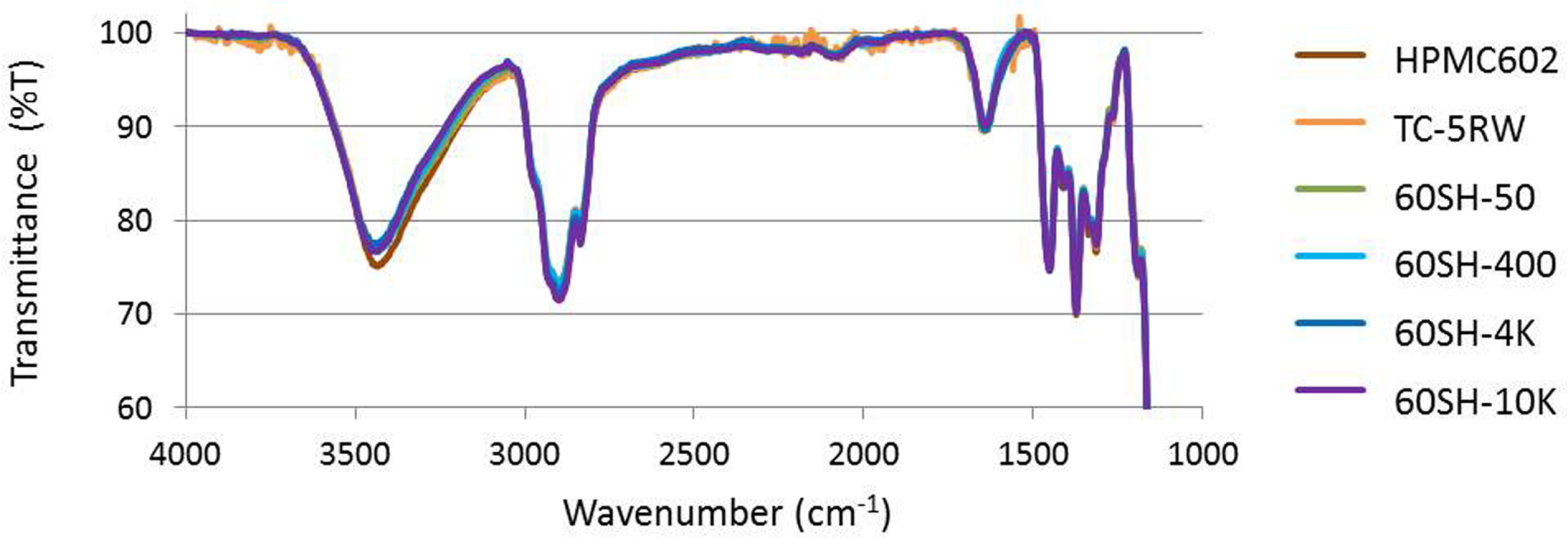
FTIR spectra of the fingerprint region of HPMCs. IR spectra were superimposed.

To analyze the difference in the characteristic vibrations of the HPMC functional groups, IR spectra were measured and superimposed. Fig 2 shows the fingerprint regions in the HPMC FTIR spectra. All spectra displayed several representative bands for the HPMCs, including 900–1300 cm^−1^, 1460 cm^−1^, around 2800 cm^−1^, and 3465 cm^−1^ for C–O–C stretches, C–H vibration, methyl group vibration, and -OH vibration, respectively. No significant differences were observed among the HPMC FTIR profiles. These results demonstrate that the chemical properties shown in Table 1 are confirmed to be common among the investigated HPMCs. Notably, spectral matching in the 1640 cm^−1^ band, which is assigned as the bending mode of water [17], was observed in all of the investigated HPMCs, which indicates that the net properties of water retention in the HPMCs were equal under our experimental conditions.

### DSC analysis

The thermal properties of 2.0 wt% aqueous solution samples of HPMCs were analyzed using DSC measurements in the temperature region 0°C–90°C. All HPMCs demonstrated endo- and exo-thermic peaks under heating and cooling processes, respectively. These thermal changes reflect transitions between aggregation and melting, which resulted from competition between polymer–polymer and polymer–solvent interactions [16, 18, 19]. This analysis is useful because the compositional characters of a polymer compound are related to the net thermal properties [20–22].

**Fig 3:**
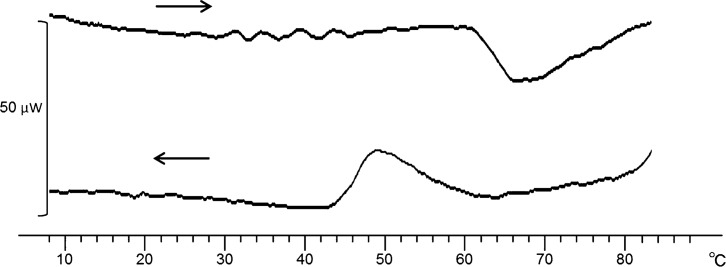
Representative thermal profile of TC-5RW. (→) and (←) indicate heating and cooling processes, respectively.

The representative thermal profile of TC-5RW is shown in Fig 3, and the results of the DSC measurements are summarized in Table 2. Small differences were observed in the endothermic peak temperatures of the HPMCs: the peak temperature of HPMC602 was a little higher and that of 60SH-10K was a little lower than that of the other HPMCs. However, no differences were observed in the HPMC exothermic peak temperatures; all were similarly located within 48°C–53°C. Although the experimental conditions were different, the observed HPMC transition temperatures in this study were consistent with those reported previously [23]. These results clearly indicate that the thermal analysis cannot detect any parameters linked to *in vivo* anti-prion activities; however, net interactions with the solvent were similar among HPMCs with a similar composition and degree of substitution, which is consistent with the interpretation of the matched intensity of the 1640 cm^−1^ band among the HPMCs in the FTIR analysis.

**Table 2:** Thermal characteristics of HPMCs.

	Heating process		Cooling process	
	Endothermic peak (°C)	ΔH (J/g)	Exothermic peak (°C)	ΔH (J/g)
HPMC602	74 ~ 78	–0.082 ~ –0.13	50 ~ 51	0.040 ~ 0.072
TC-5RW	66 ~ 68	–0.20 ~ –0.22	48 ~ 50	0.14 ~ 0.19
60SH-50	66 ~ 70	–0.023 ~ –0.60	51 ~ 53	0.015 ~ 0.020
60SH-400	66 ~ 68	–0.20 ~ –0.35	50 ~ 53	0.045 ~ 0.073
60SH-4K	66 ~ 68	–0.34	48 ~ 50	0.16
60SH-10K	63 ~ 64	–0.27 ~ –0.39	51 ~ 53	0.058 ~ 0.11

### Pyrene conjugation and spectroscopic analysis

As described, no differences were observed among the six different-sized HPMCs in the overall chemical or thermal properties. HPMCs are intrinsically heterogeneous in terms of their chain length and modification at the monomer level. Therefore, we investigated whether there is an inter- or intra-molecular local environmental difference among HPMCs using the characteristic fluorescence properties of pyrene [24, 25]. Usually, in a study on the transition processes of aggregation states using unconjugated pyrene [26–28], data are obtained as an ensemble of solvatochromism of pyrene molecules interacting with other pyrene molecules, polymer molecules, and bulk solvent molecules. To extract the responses from the local environments of HPMCs, pyrene conjugation [29, 30] was performed on the HPMCs. The yields of pyrene-conjugated HPMCs, which were calculated from the resulting powder materials of the repeated precipitation and solubilization steps, were 23%, 64%, 82%, 51%, 74%, and 70% for HPMC602, TC-5RW, 60SH-50, 60SH-400, 60SH-4K, and 60SH-10K, respectively.

**Fig 4:**
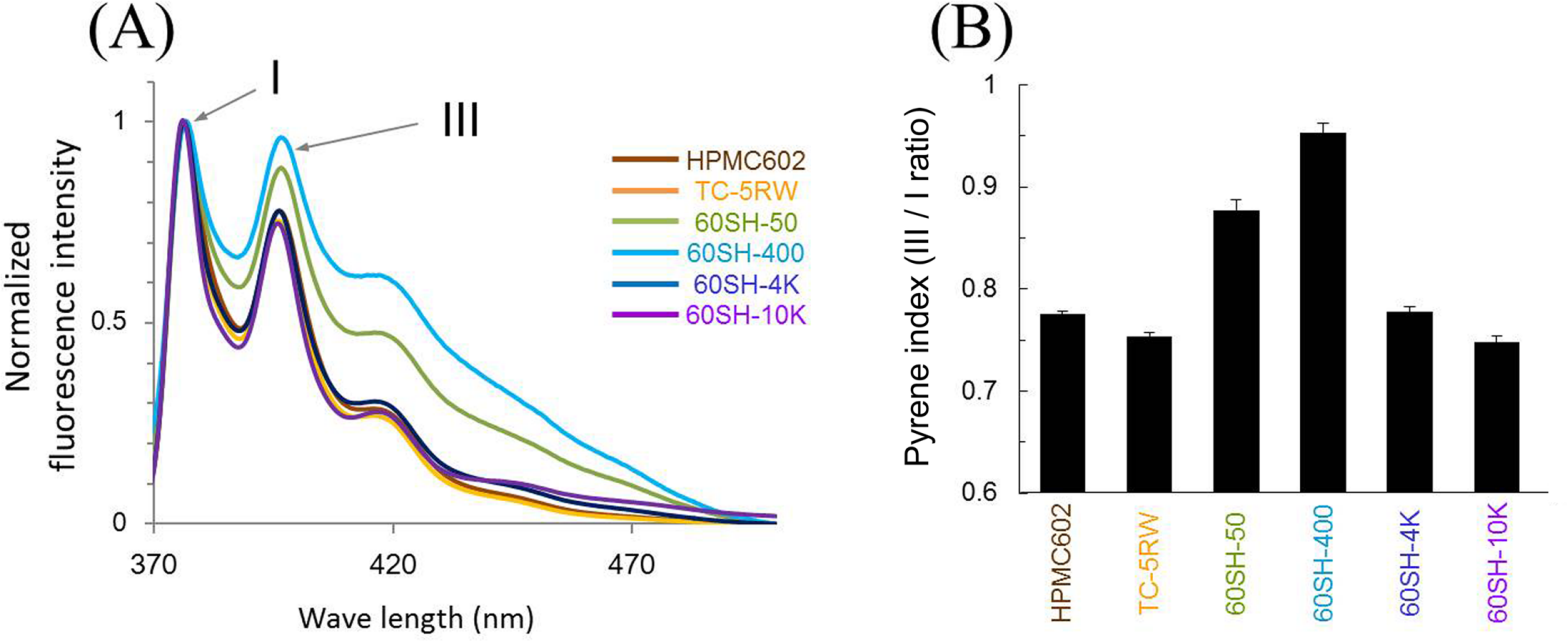
Pyrene conjugation and spectroscopic analysis of HPMCs. (A) Fluorescence spectra of pyrene-conjugated HPMCs. The fine structures of peaks indicated by I and III are clearly observed, and no excimer emission of pyrene is observed around 460 nm. Spectra were normalized by the intensity of peak I and superimposed. (B) Pyrene index (intensity ratio of peak III/peak I) of pyrene-conjugated HPMCs. The mean and standard deviation of triplicate experiments are shown.

Fig 4A shows the fluorescence spectra of pyrene-conjugated HPMCs. The fine structures of the steady-state fluorescence spectra were clearly detected, as indicated by peaks I and III. In addition, no excimer emission of pyrene–pyrene interaction was observed around 460 nm, which might be because pyrene conjugation occurred at a very low level in type E HPMCs (degree of substitution, 2.1; Table 1) and because these HPMCs have fewer hydroxyl groups available for pyrene conjugation than other HPMCs such as types F (2906) and K (2208) [10].

The fine structure comprises the vibrational variations in pyrene. Because vibronic band intensities are perturbed by specific solute–solvent dipole–dipole coupling and the energy gap of the electronic state, it has been demonstrated that the fluorescence intensity ratio of peak III/peak I (hereinafter referred to as the pyrene index) represents the dielectric properties around pyrene, specifically, local hydrophobic environments [31]. Fig 4B shows the HPMC pyrene indices, which were distributed over 0.75–0.95. Interestingly, these values were similar to those of simple polar solvents, such as methanol (0.75), ethanol (0.91), and *n*-propanol (0.92) [31, 32], suggesting that the pyrene-conjugated molecules are surrounded by various methyl and hydroxypropyl groups and that the pyrene indices reflect the local environments of HPMCs.

**Fig 5:**
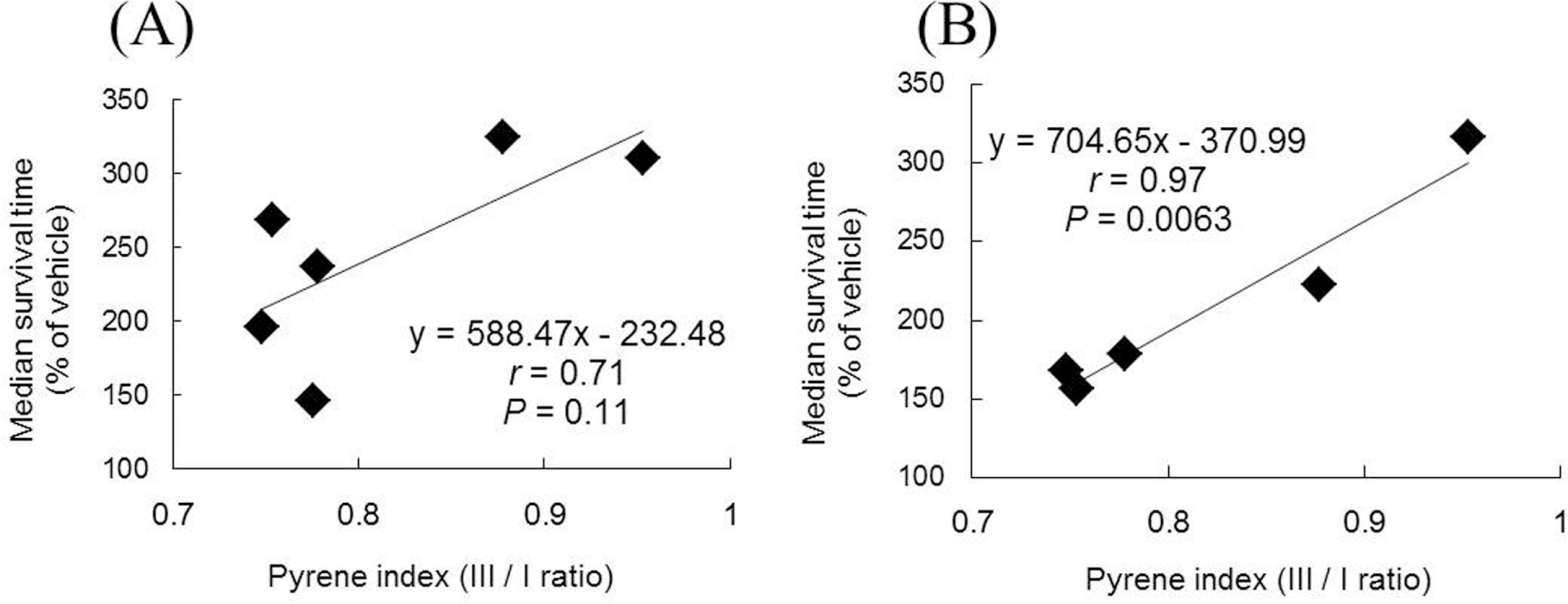
Relationships of the pyrene index with the *in vivo* median survival time of HPMCs. The data of Fig 4B are compared with those of the median survival time in the immediate post-infection treatment (A) or with those in the 1-year pre-infection treatment (B), which are presented in Table 1.

We investigated the relationship between the pyrene index and the *in vivo* anti-prion activity of HPMCs. The results are shown in Fig 5; only a moderate correlation (r = 0.71, P = 0.11) was observed between the pyrene index and anti-prion activity in the immediate post-infection treatment (Fig 5A), whereas a significant correlation (r = 0.97, P = 0.0063) was observed in the 1-year pre-infection treatment (Fig 5B); thus, there is a convergence of the relationship in an administration timing-dependent manner, which suggests that the pyrene index denoting long-lasting efficacy of HPMCs may be related to the retention of HPMCs in the body.

### Pyrene index and macrophage uptake level

**Fig 6:**
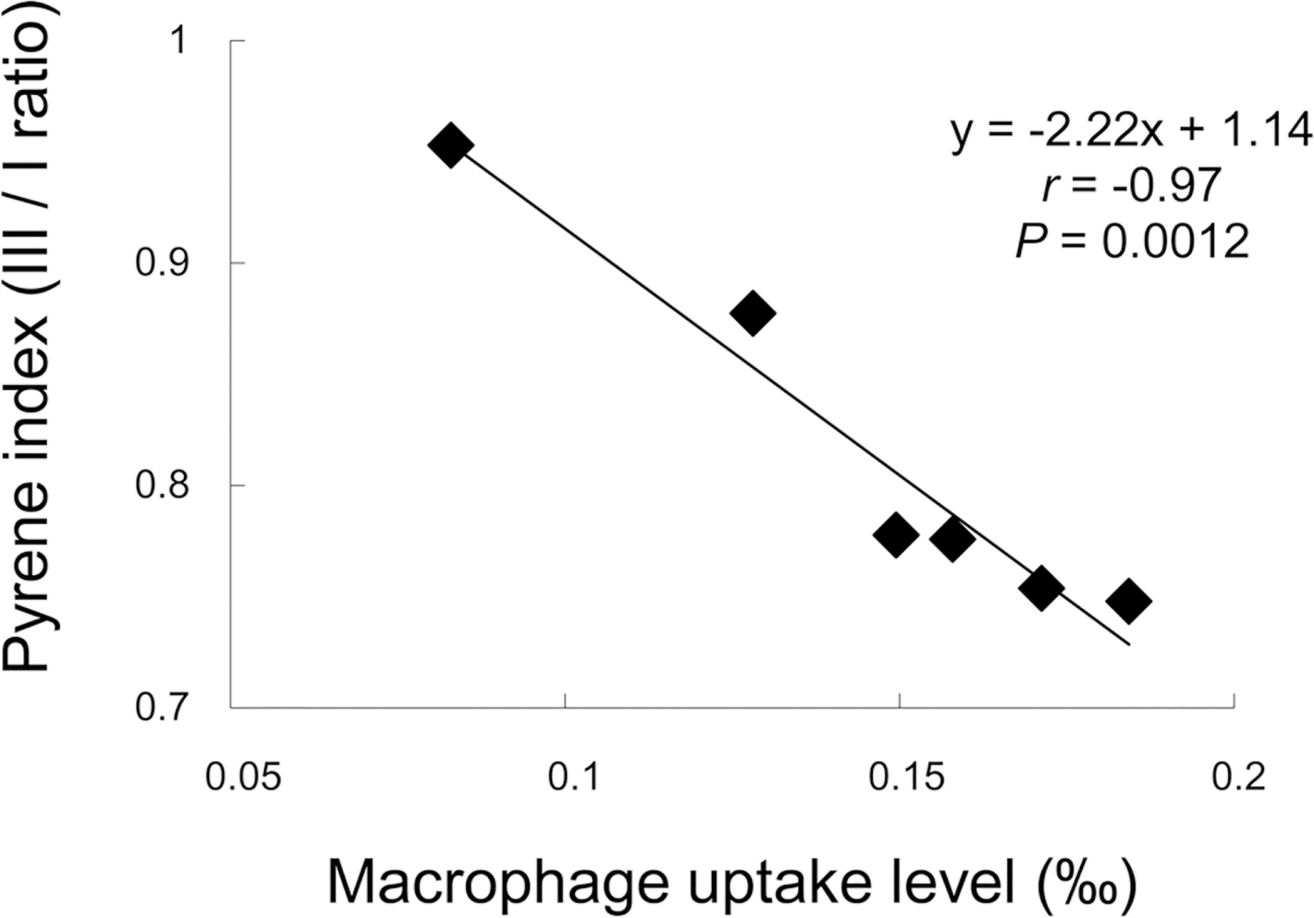
Relationships of the pyrene index with the macrophage uptake level of HPMCs. The data of Fig 4B are compared with those of the macrophage uptake level, which are presented in Table 1.

Previously, we have reported that the macrophage uptake level of HPMCs is negatively correlated with anti-prion activity, especially in the 1-year pre-infection treatments [9]. This correlation was negative but very similar to that of the pyrene index. Thus, the relationship of the pyrene index with the HPMC macrophage uptake level was investigated. As shown in Fig 6, the pyrene index significantly and negatively correlated with the macrophage uptake level (r = −0.97, P = 0.0012). Since it has been reported that the macrophage affinity to or macrophage uptake of nanoparticles depends on the surface hydrophobicity of the nanoparticles [33–36], we investigated whether HPMCs with higher pyrene indices have fewer hydrophobic surfaces by measuring the ζ-potentials. However, the ζ-potentials of HPMCs were around zero and there were no obvious differences.

These results indicate that elevation of the local hydrophobic environments of the HPMC molecules leads to a decrease in the macrophage uptake level, regardless of the overall charge on the surface of HPMC molecules. It is unclear what kinds of local hydrophobic environments are represented by the pyrene index and how they are inhibitory to macrophage uptake. The fine structure of local hydrophobic environments denoted by the pyrene index as well as the mechanism of their interaction with macrophages needs to be elucidated in the future.

### Distribution of HPMCs *in vivo*

According to our previous study on the pharmacokinetics of ^14^C-labeled TC-5RW [9], subcutaneously-administered TC-5RW is rapidly taken into the blood and excreted in the urine and feces at a rate of 70% of the initial dose within 3 days, and thereafter at a considerably slower rate. Radiolabeled TC-5RW is soon distributed throughout the body and stays in tissues or fluids, with the least amount in brain parenchyma and the most abundant quantities in tissues or fluids, such as adrenal cortex, brain choroid plexus, mandibular and thyroid glands, lymph, spleen, and skin, with an elimination half-life in these tissues or fluids of 50–350 days.

Therefore, we analyzed tissue distribution using fluorescein-labeled HPMCs in two representative tissues (brain choroid plexus and spleen) in mice. The brain choroid plexus was selected because it is located in the center of the brain, which is the primary organ affected by prions, while the spleen was selected because it is one of the peripheral tissues where prions abundantly accumulate in amounts close to those in the brain [37–40].

As a preliminary step, we examined the rationale of using fluorescein-labeled HPMCs *in vivo* by comparing the data of fluorescein-labeled TC-5RW with the previous data of radiolabeled TC-5RW. The amount of fluorescein-labeled TC-5RW in the blood and plasma of ddY mice at 1 week after intraperitoneal injection was 42 μg/mL and 72 μg/mL, respectively, which was within a similar dose range for radiolabeled TC-5RW in blood (approximately 100 μg/mL [9]) of Tg7 mice at 1 week after subcutaneous injection. Although the experimental conditions were different, these findings suggest that using fluorescein-labeled HPMCs *in vivo* is feasible.

**Fig 7:**
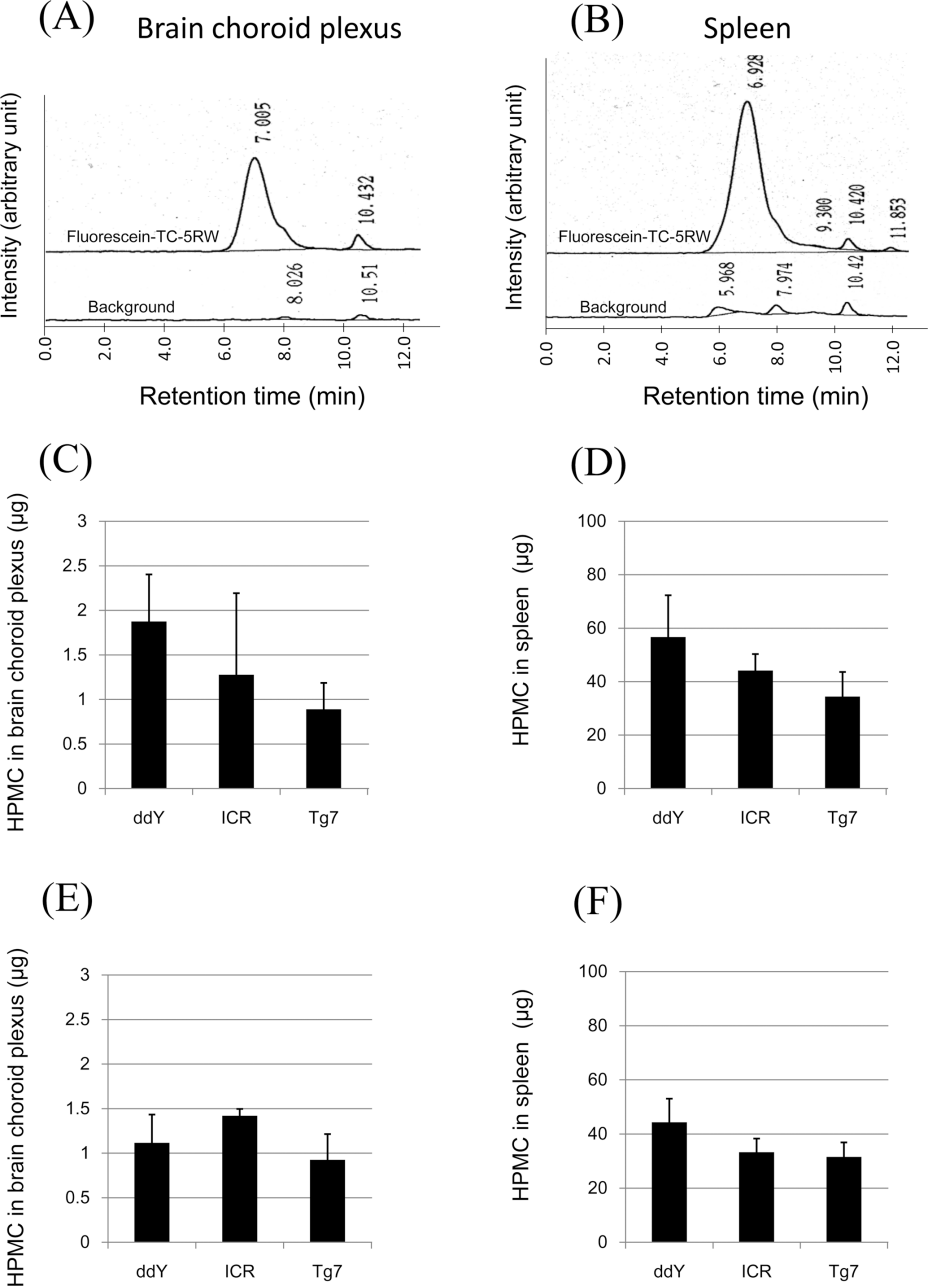
Distribution levels in brain choroid plexus or spleen of mice intraperitoneally injected with HPMCs. Representative GPC profiles of fluorescein-labeled TC-5RW are shown for brain choroid plexus (A) and spleen (B) of ddY mice at 1 week after intraperitoneal injection. The distribution levels of fluorescein-labeled TC-5RW are shown for brain choroid plexus (C, E) and spleen (D, F) of ddY, ICR, and Tg7 mice at 1 week (C, D) or 8 weeks (E, F) after intraperitoneal injection. It is noted that there is no significant difference among the three mouse strains.

Fig 7 shows the representative GPC profiles of fluorescein-labeled TC-5RW in brain choroid plexus (Fig 7A) and spleen (Fig 7B) of ddY mice at 1 week after intraperitoneal injection. From the standard curves of this compound, the peak areas were found to correspond to 0.11 μg and 0.26 μg of the compound in brain choroid plexus and spleen, respectively. Thus, the detection of fluorescein-labeled HPMCs in tissues is feasible at a sub-μg level.

Since ddY and ICR mice are commonly used in pharmacokinetic studies and toxicity studies, both strains of mice as well as Tg7 mice were examined for the distribution level of fluorescein-labeled TC-5RW in brain choroid plexus and spleen at 1 week or 8 weeks after intraperitoneal injection. As shown in Fig 7C–7F, there was no significant difference among these mice. Therefore, ddY mice were used for further analysis, because our previous study demonstrated HPMC effectiveness in ddY mice as well as Tg7 mice, but not in ICR mice [9], and because ddY mice are easier to handle than Tg7 mice.

### Distribution level and anti-prion activity

**Fig 8:**
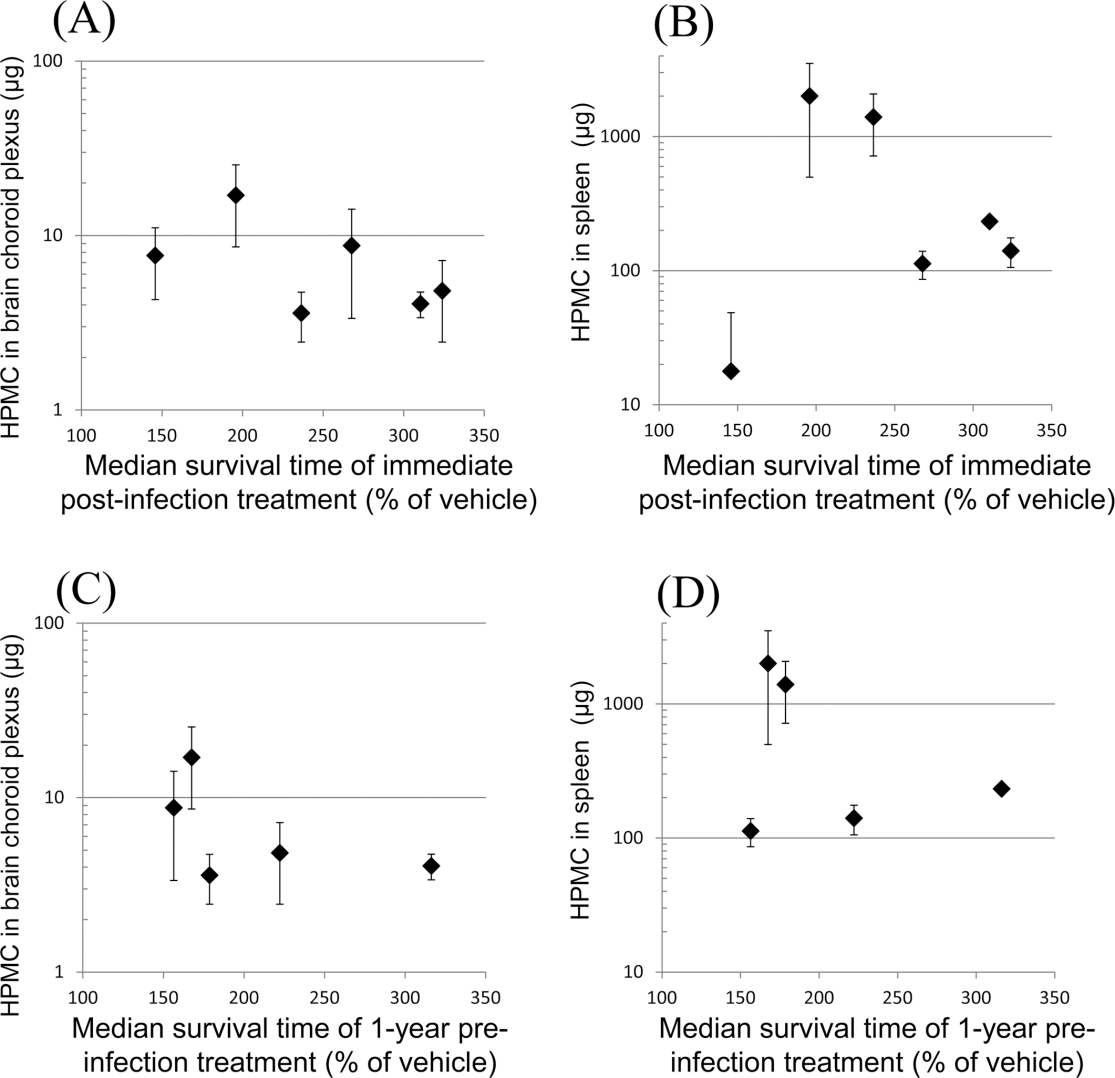
Relationship of the distribution level with the anti-prion activity. The distribution levels of fluorescein-labeled HPMCs in brain choroid plexus (A, C) or spleen (B, D) of ddY mice are plotted against the anti-prion activities of the immediate post-infection (A, B) or of the 1-year pre-infection (C, D) treatments, which are presented in Table 1.

The distribution levels of fluorescein-labeled HPMCs in brain choroid plexus and spleen of ddY mice were compared with the anti-prion activities in Tg7 mice previously reported [9]. Fig 8A (brain choroid plexus) and 8B (spleen) show the relationship of the distribution level with the anti-prion activity of the immediate post-infection treatment, while Fig 8C (brain choroid plexus) and 8D (spleen) show the relationship with the anti-prion activity of the 1-year pre-infection treatment. The findings demonstrate that the tissue distribution levels do not correlate with the *in vivo* anti-prion activities. However, it is still possible that prion infection may affect the distribution levels in tissues. This possibility could not be evaluated in the present study because of biosafety issues and remains to be evaluated in the future.

### Distribution level and molecular weight

**Fig 9:**
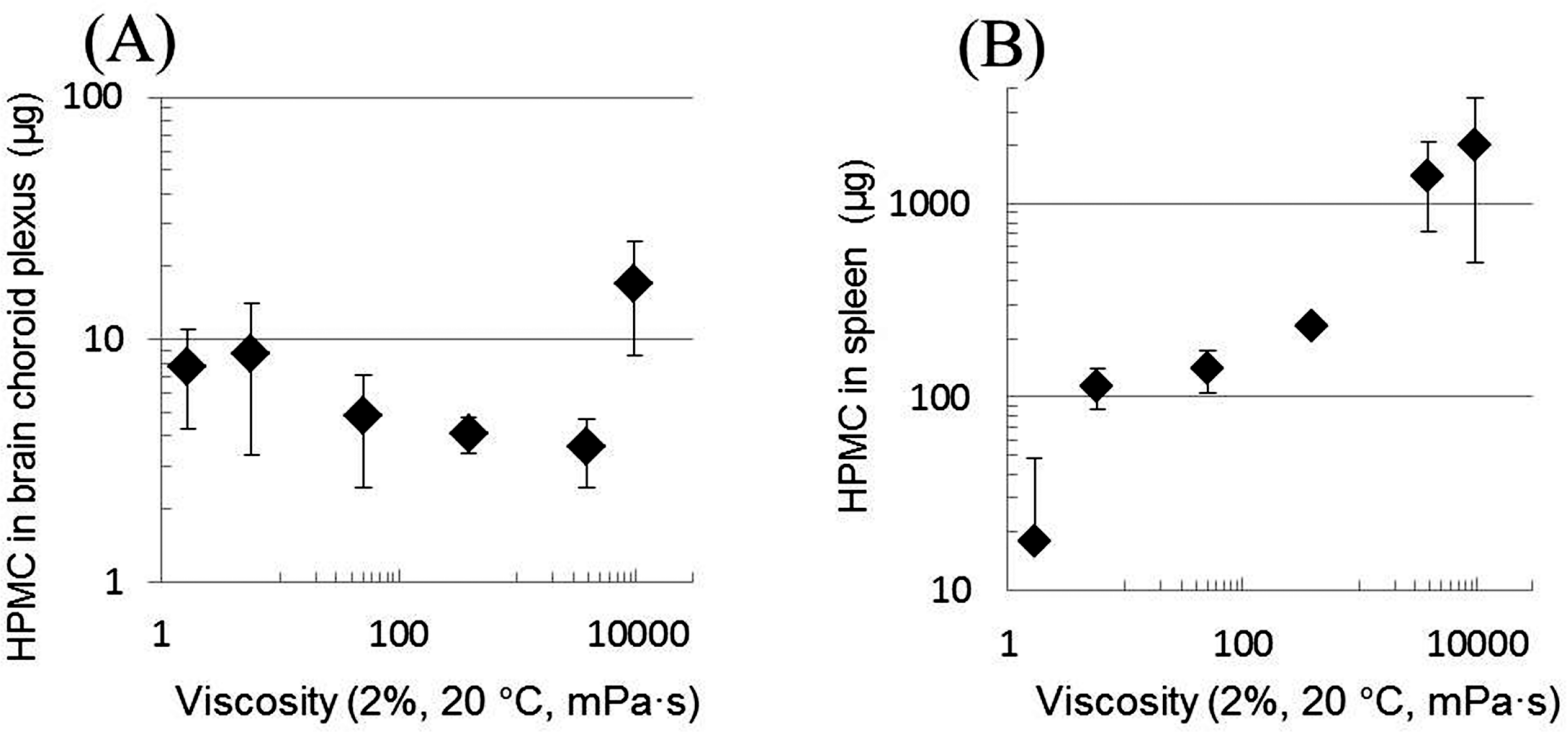
Relationship between the distribution and the molecular weight of HPMCs. The distributions in brain choroid plexus (A) or spleen (B) are plotted against the *M*_n_ of HPMCs in Table 1.

As shown in Fig 9A (brain choroid plexus) and 9B (spleen), the distribution in brain choroid plexus was independent of the molecular weight of the HPMCs; however, the distribution in spleen was apparently proportional to the molecular weight of the HPMCs. These results suggest that the HPMC distribution pattern is not identical even in abundantly distributed tissues in the body. In addition, these results suggest that larger molecular weight HPMCs are likely to be trapped in the spleen, which may be similar to methylcellulose compounds that are reported to be trapped in the spleen, thereby causing splenomegaly and hypersplenism [41]. However, the weights of the spleens of the mice were within 0.10–0.21 g in the present study, and no significant relationships were observed between spleen weight and molecular weights of HPMCs.

Since the distribution level varies from tissue to tissue, the distribution level of brain parenchyma, which is the target of prion disease, may be important for elucidating the relationship with anti-prion activity. However, in the present study using fluorescent labeled HPMCs, the distribution level in brain parenchyma could not be evaluated because of limitations in the measurement sensitivity.

## Conclusions

Even in HPMCs with similar composition and degree of substitution, pyrene conjugation and spectroscopic analysis successfully detected chemical structural differences that correlated with *in vivo* anti-prion activities, which could not be detected by other chemical or tissue distribution analyses. Interestingly, the pyrene indices of these HPMCs negatively correlated with the *ex vivo* macrophage uptake ratios of HPMCs. However, the mechanism for these correlations remains unclear. The structural nature of the local dielectric differences on HPMCs, as denoted by the pyrene index, is also an enigma. Nevertheless, the present study indicates that pyrene conjugation and spectroscopic analysis are useful and sensitive methods for detecting biologically meaningful chemical differences in the polymers that comprise heterogeneous molecules with similar composition and degree of substitution.
